# Silencing of *GhSHP1* hindered flowering and boll cracking in upland cotton

**DOI:** 10.3389/fpls.2025.1558293

**Published:** 2025-02-25

**Authors:** Wenjuan Xu, Qi Ma, Jisheng Ju, Xueli Zhang, Wenmin Yuan, Han Hai, Caixiang Wang, Gang Wang, Junji Su

**Affiliations:** ^1^ State Key Laboratory of Aridland Crop Science, College of Life Science and Technology, Gansu Agricultural University, Lanzhou, China; ^2^ Key Laboratory of Cotton Genetic Improvement and High-Yield Cultivation, Xinjiang Production and Construction Corps, and Cotton Research Institute, Xinjiang Academy of Agricultural and Reclamation Sciences, Shehezi, Xinjiang, China

**Keywords:** *Gossypium hirsutum*, cotton boll cracking, GhSHP1, virus-induced gene silencing (VIGS), paraffin section

## Abstract

The opening of cotton bolls is an important characteristic that influences the precocity of cotton. In the field, farmers often use chemical defoliants to induce cotton leaves to fall off earlier, thus accelerating the cracking of cotton bolls. However, the molecular mechanism of cotton boll cracking remains unclear. We identified ten *AGAMOUS* subfamily genes in upland cotton. Three pairs of *Gossypium hirsutum* AG subfamily genes (*GhAGs*) were amplified via tandem duplication. The promoters of the *GhAGs* contained a diverse array of *cis*-acting regulatory elements related to light responses, abiotic stress, phytohormones and plant growth and development. Transcriptomic analyses revealed that the expression levels of *GhAG* subfamily genes were lower in vegetative tissues than in flower and fruit reproductive organs. The qRT−PCR results for different tissues revealed that the *GhSHP1* transcript level was highest in the cotton boll shell, and *GhSHP1* was selected as the target gene after comprehensive analysis. We further investigated the functional role of *GhSHP1* using virus-induced gene silencing (VIGS). Compared with those of the control plants, the flowering and boll cracking times of the *GhSHP1*-silenced plants were significantly delayed. Moreover, the results of paraffin sectioning at the back suture line of the cotton bolls revealed that the development of the dehiscence zone (DZ) occurred later in the *GhSHP1*-silenced plants than in the control plants. Furthermore, at the same developmental stage, the degree of lignification in the silenced plants was lower than that in the plants transformed with empty vector. The expression of several upland cotton genes homologous to key *Arabidopsis* pod cracking genes was significantly downregulated in the *GhSHP1*-silenced plants. These results revealed that *GhSHP1* silencing delayed the flowering and cracking of cotton bolls and that the cracking of cotton bolls was delayed due to effects on DZ development. These findings are highly important for future studies of the molecular mechanism of cotton boll cracking and for breeding early-maturing and high-quality cotton varieties.

## 1 Introduction

The fruits of many flowering plants (such as siliques, capsules, and pods) need to crack and release their seeds after maturity to support the reproduction of future generations. The cracking of the fruit after ripening is called pod shattering in soybeans, silique dehiscence in *Brassica napus*, and boll opening in upland cotton. Although seed dispersal through fruit cracking is an important method of plant reproduction, the application of this trait is completely different in different crop breeding methods. For crops such as soybeans and oilseed rape, the premature cracking of pods or siliques can easily lead to seed shattering, resulting in a significant reduction in crop yield. However, the cracking of cotton bolls is conducive to the release of cotton fibers, and early boll opening is one of the key target traits of cotton breeding for early maturity. Although many studies have explored the mechanism of silique and pod cracking (Liljegren et al., 2000), the mechanism of capsule cracking (such as in cotton bolls) has not been studied extensively.

The mechanism of silique dehiscence has been explored mainly in the model plant *Arabidopsis thaliana*. When the silique shell of *A. thaliana* is transected, three components can be observed in the silique shell: the valves, valve margins and replum (Dinneny and Yanofsky, 2005; Chen et al., 2024). Valves develop from the carpel, which consists of six layers of cells, including the outer epidermis, three layers of mesophyll tissue, endocarp layer *b* (en*b*) and endocarp layer *a* (en*a*) (degenerate during fruit ripening) (Dinneny and Yanofsky, 2005; Gu et al., 1998; Vivian-Smith et al., 2001). The area between the valve and the replum is called the valve margin and is composed of a lignified layer (LL) and a separation layer (SL). These two layers also form dehiscence zones (DZs) during silique cracking (Ferrándiz et al., 1999). A gene regulatory network involved in silique cracking initially forms (Ballester and Ferrándiz, 2017). Studies of this regulatory network revealed that the MADS-box (MCM1, AGAMOUS, DEFICIENS, SRF) transcription factors *SHATTERPROOF1* (*SHP1/AGL1*) and *SHATTERPROOF2* (*SHP2/AGL5*) (Liljegren et al., 2000) and the basic helix-loop-helix (bHLH) transcription factors *INDEHISCENT* (*IND*) (Liljegren et al., 2004) and *ALCATRAZ* (*ALC*) (Rajani and Sundaresan, 2001) are associated with the development of the valve margin and regulate the development of the DZ, leading to silique cracking (Ferrándiz, 2002; Lewis et al., 2006). As part of this regulatory pathway, *SHP1/2* positively regulate the downstream factors *IND* and *ALC*. Moreover, *IND* and *ALC* control valve margin development independently of each other. *SHP1/2* and *IND* are essential for the development of both the lignified layer and the separation layer, whereas *ALC* is required for the formation of only the separation layer (Liljegren et al., 2000, 2004; Rajani and Sundaresan, 2001). The activities of valve margin identity genes are inhibited by *FRUITFULL* (*FUL*) in the valve (Ferrándiz et al., 2000; Gu et al., 1998) and the transcriptional regulatory factor *REPLUMLESS* (*RPL*), which is involved in the development of the replum (Ferrándiz et al., 2000; Roeder et al., 2003; Liljegren et al., 2004). In other words, *SHP1/2*, *IND* and *ALC* expression is limited to the valve margin through repression by *FUL* in the valve and by *RPL* in the replum. *AP2* negatively regulates the expression of replum and valve margin identity genes to prevent excessive growth of replum and valve margins (Ripoll et al., 2011). *NAC SECONDARY WALL THICKENING PROMOTING FACOTR1* (*NST1*), which is expressed in the en*b* layer and highly expressed in developing LL cells, modulates silique cracking by controlling cell wall thickening (Mitsuda and Ohme-Takagi, 2008; Zhong et al., 2010). *ARABIDOPSIS DEHISCENCE ZONE POLYGALACTURONASE1* (*ADPG1*), which is expressed in the SL in DZs, encodes plant-specific endo-polygalacturonases (PGs) and promotes silique cracking by reducing cell adhesion (Ogawa et al., 2009; Roberts et al., 2002). In addition, some plant hormones, such as auxin, cytokinin and gibberellin, regulate the development of siliques, pods and capsules, and ethylene is a growth regulator that is commonly used to promote cotton boll cracking (Arnaud et al., 2010; Forlani et al., 2019; Larsson et al., 2014; Marsch-Martínez et al., 2012; Simonini et al., 2017; Zuñiga-Mayo et al., 2018).

As an important breeding trait for early maturity in upland cotton, early boll opening has long been a focus of breeders. Farmers use chemical defoliating agents in field plantings to induce cotton leaves to fall off early and accelerate cotton boll opening (Li et al., 2022). Moreover, although cotton bolls are a typical type of capsule, the mechanism by which they crack remains unclear. In this study, the *AG* subfamily genes of upland cotton were first identified; then, family analysis was performed using bioinformatics methods. The tissue-specific expression patterns of *Gossypium hirsutum* AG subfamily genes (*GhAGs*) were analyzed on the basis of transcriptome data from the upland cotton variety TM-1, and the tissue of Zhongmian113 was used as a template to analyze the expression patterns of *GhAGs* using qRT−PCR. To clarify the role of *GhSHP1/AGL1* in upland cotton, we used virus-induced gene silencing (VIGS) technology to reduce *GhSHP1* transcript levels. Phenotypic changes paraffin sections of cotton boll shells at different developmental stages, and *GhIND*, *GhFUL*, *GhALC*, *GhRPL* and *GhNST1* expression were observed and analyzed in the *GhSHP1*-silenced plants and controls. These findings increase our understanding of *GhSHP1* and provide a theoretical basis for future research on the cracking mechanism of cotton bolls and for the cultivation of early-maturing and high-quality cotton varieties.

## 2 Materials and methods

### 2.1 Identification and characteristics of *AGAMOUS* subfamily genes in upland cotton

The *AGAMOUS* subfamily in the model plant *Arabidopsis thaliana* contains four genes: *AGAMOUS* (*AG*, AT4G18960) (Bowman et al., 1989; Yanofsky et al., 1990), *SHATTERPROOF1* (*SHP1/AGL1*, AT3G58780), *SHATTERPROOF2* (*SHP2/AGL5*, AT2G42830) (Liljegren et al., 2000; Ma et al., 1991) and *SEEDSTICK* (*STK/AGL11*, AT4G09960) (Pinyopich et al., 2003; Rounsley et al., 1995). The protein sequences of these four genes in *A. thaliana* were obtained from The Arabidopsis Information Resource website (TAIR, https://www.arabidopsis.org) as seed sequences. The AG subfamily protein sequences of 14 plant species (**Supplementary Table S1**) were obtained using BLAST searches and downloaded from the Phytozome website (https://phytozome-next.jgi.doe.gov/) and the Ensembl Plants website (https://plants.ensembl.org/index.html), with the expected value set to < *e* ^-50^. The protein physicochemical properties of the *GhAG* subfamily genes were analyzed using the ExPASy website (https://www.ExPASy.org/). The subcellular localizations of *GhAG* subfamily genes were predicted using the WOLF PSORT website (https://wolfpsort.hgc.jp/).

### 2.2 Phylogenetic and collinearity analysis of *AG* subfamily genes

We conducted multiple alignments of AG subfamily protein sequences from 15 plant species using the ClustalW tool, and a phylogenetic tree was constructed using the neighbor-joining (NJ) method with 1000 replications and the *p* distance method with pairwise deletions using MEGA 7.0 software (de Moura et al., 2017; Kumar et al., 2016). Visualization and beautification were then performed using the iTOL website (https://itol.embl.de/) (Letunic and Bork, 2019). The genomes and annotated files of *Gossypium hirsutum*, *Gossypium raimondii* and *Gossypium arboretum* are available on the Ensembl Plants website (https://plants.ensembl.org/index.html). Then, intraspecific collinearity analysis of upland cotton and interspecific collinearity analysis of the three *Gossypium* species were performed with TBtools (v.2.091) software, and the synonymous (*Ks*) and nonsynonymous (*Ka*) substitution rates of the upland cotton *AG* subfamily genes were calculated (Chen et al., 2020; Luo et al., 2024).

### 2.3 Analysis of *cis*-acting elements in the promoters of *GhAGs*

The sequences of the 2000-bp regions located upstream of the *GhAG* genes were obtained from CottonFGD (https://cottonfgd.net/) and utilized to identify *cis*-regulatory elements associated with *GhAG* genes through the PlantCARE program (http://bioinformatics.psb.ugent.be/webtools/plantcare/html/). Excel software was employed to visualize the results derived from these predictions.

### 2.4 Analysis of the expression patterns of *GhAG* subfamily genes

The expression patterns of *AG* subfamily genes in upland cotton were analyzed using RNA-seq data obtained from 13 distinct tissues of the upland cotton variety TM-1 (http://cotton.zju.edu.cn/). Gene expression levels were quantified based on fragments per kilobase of transcript per million mapped fragments (FPKM) values. Heatmaps depicting the expression patterns of *GhAG* subfamily genes were generated utilizing TBtools software.

### 2.5 Plant materials

Leaf rot soil and vermiculite were mixed 1:1 in a seedling cup and soaked until the surface was slightly wet. Intact seeds were selected and planted 1.5 cm below the soil. The artificial climate incubators were established with fixed conditions (16 h light/8 h darkness, 25°C, and 70% humidity) for growing the seedlings. The roots, stems, leaves, sepals, petals, ovaries, cotton shells and cotton fibers were sampled in the 1st, 4th to 5th and 11th to 17th weeks of cotton growth. Early opening bolls (Yuzhishi84-1) and late opening bolls (Ganmain12) varieties were planted, and cotton boll shell tissues from different development stages were collected. The samples were quickly frozen in liquid nitrogen and stored at -80°C for future use.

### 2.6 Quantitative real-time PCR analysis

Total RNA was extracted utilizing an AFTSpin Complex Plant Fast RNA Extraction Kit (No. RK30122; ABclonal, China). The concentration, purity and integrity of the RNA were assessed using RNA electrophoresis and an ultramicro concentration detector. Reverse transcription was conducted utilizing a UnionScript First-Strand cDNA Synthesis Mix Kit (No. SR511; Genesand, China). Specific primers for five *GhAGs* were designed using Primer-BLAST (https://www.ncbi.nlm.nih.gov/tools/primer-blast/index.cgi) for qRT−PCR. The qRT−PCR assay was conducted utilizing BrightCycle Universal SYBR Green qPCR Mix with a UDG kit (No. RK21219; ABclonal, China) on a LightCycler^®^ 96 Instrument (Roche, Switzerland). *GhACTIN* (Zhao et al., 2020) was utilized as an internal reference gene for qRT−PCR for normalization of transcript levels. The relative expression level of each gene was determined using the 2^−ΔΔ^ *^CT^* method. Three biological replicates were performed to ensure accuracy and reliability. Statistical analysis was conducted using SPSS 27 software (IBM, Armonk, NY, USA). The least significant difference (LSD) test was employed to calculate *P* values to assess significance (Rao et al., 2013; Willems et al., 2008; Zhao et al., 2020).

### 2.7 Virus-induced *GhSHP1* silencing in upland cotton

*GhSHP1* silencing was achieved with VIGS technology using a CLCrV carrier. The 463-bp target fragment of *GhSHP1* was obtained using PCR amplification and integrated into the CLCrV vector. The recombinant plasmid was subsequently introduced into *Agrobacterium tumefaciens* strain GV3101. The transformed *A. tumefaciens* was then resuspended. After three hours in the dark, CLCrV: *ChlI*, the empty vector and CLCrV: *GhSHP1* were mixed with the auxiliary bacteria at a ratio of 1:1. Finally, the mixed bacterial mixture was injected into the dorsal surface of the cotyledons of 7-day-old plants to produce *GhSHP1*-silenced cotton plants (CLCrV: *GhSHP1*), along with negative control (empty vector) and positive control (CLCrV: *ChlI*) plants. The plants were subjected to a 24-h incubation period in darkness prior to being cultured under standard conditions. When the leaves of the positive control plants were yellow, the target gene had been successfully silenced. The seedlings were subsequently transplanted into flowerpots for further cultivation. At 15 and 3 days postanthesis (DPA), the cotton boll shells were sampled as templates, and *GhALC*, *GhFUL*, *GhIND*, *GhNST1* and *GhRPL* expression levels were detected using qRT−PCR.

### 2.8 Microscopic observation of boll shell anatomy

Cotton boll shells were sampled at 0, 3, 5, 10 and 15 DPA and stored in FAA fixative. After the samples were fixed for more than 24 hours, they were dehydrated, dipped in wax, and embedded to generate paraffin sections (4 μm). The paraffin sections were subjected to staining with safranin O solution and plant solid green staining solution, followed by mounting in neutral balsam. The sections were observed using a Nikon Eclipse E100 microscope (Nikon, Japan), and images were acquired with a Nikon DS-U3 system.

## 3 Results

### 3.1 Identification and physicochemical properties of *AGAMOUS* subfamily genes in upland cotton

**Figure 1:**
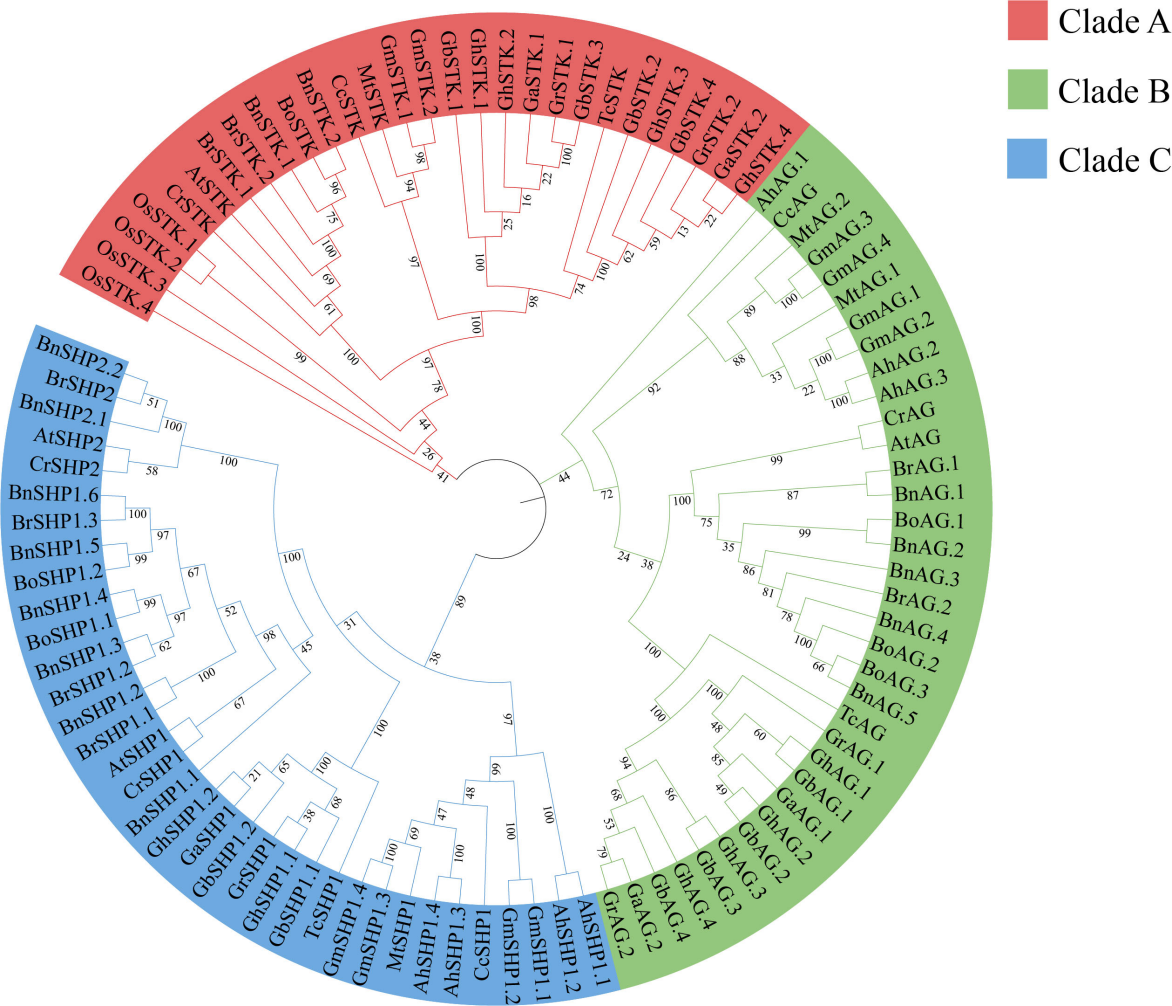
Evolutionary relationships of the AG subfamilies in 15 species. Gh, Gb, Gr, Ga, At, Bn, Br, Bo, Cr, Gm, Ah, Mt, Cc, Os and Tc represent Gossypium hirsutum, G. barbadense, G. raimondii, G. arboreum, Arabidopsis thaliana, Brassica napus, B. rapa ssp. pekinensis, B. oleracea capitata, Capsella rubella, Glycine max, Arachis hypogaea, Medicago truncatula, Cercis canadensis, Oryza sativa and Theobroma cacao, respectively.

In this study, we identified ten *AGAMOUS* (*AG*) subfamily genes in upland cotton. On the basis of their homology with *Arabidopsis thaliana AG* subfamily genes in the phylogenetic tree, these genes were named *GhAG.1*, *GhAG.2*, *GhAG.3*, *GhAG.4*, *GhSHP1.1*, *GhSHP1.2*, *GhSTK.1*, *GhSTK.2*, *GhSTK.3* and *GhSTK.4* (**Supplementary Table S1**, **Figure 1**). The lengths and molecular weights of the ten GhAG proteins exhibited minimal variation, ranging from 223 to 246 amino acids (aa) and from 25.72 to 28.37 kDa, respectively. The isoelectric points of the ten GhAG proteins varied between 9.25 and 9.44, indicating that these proteins are alkaline. The instability index and average hydropathicity of the GhAG proteins varied from 48.47 to 62.44 and from -0.848 to -0.601, respectively. These results suggest that the *GhAGs* encode unstable hydrophilic proteins. Subcellular localization predictions suggested that these proteins were localized within the nucleus.

### 3.2 Phylogenetic analysis of *AG* subfamily members

To clarify the evolutionary relationships among the *AG* subfamily genes, a total of 98 *AG* subfamily genes (**Supplementary Table S2**) from 15 different species were employed to construct a phylogenetic tree. The phylogenetic tree was classified into three main branches (**Figure 1**). The numbers of genes contained in clades A (n= 28, 28.57%), B (n=35, 35.71%) and C (n=35, 35.71%) were similar. Among the ten *GhAG* genes, four, four and two *GhAGs* clustered with *AtAG*, *AtSTK/AGL11*, and *AtSHP1/AGL1*, respectively. However, no homologs of *AtSHP2/AGL5* were found among the *GhAGs*, *GbAGs*, *GaAGs* or *GrAGs*. Among the other ten species, genes homologous to *AtSHP2* were found in only three silique species (*Bn*, *Br* and *Cr*).

### 3.3 Gene duplication of the AG subfamily in cotton species

**Figure 2:**
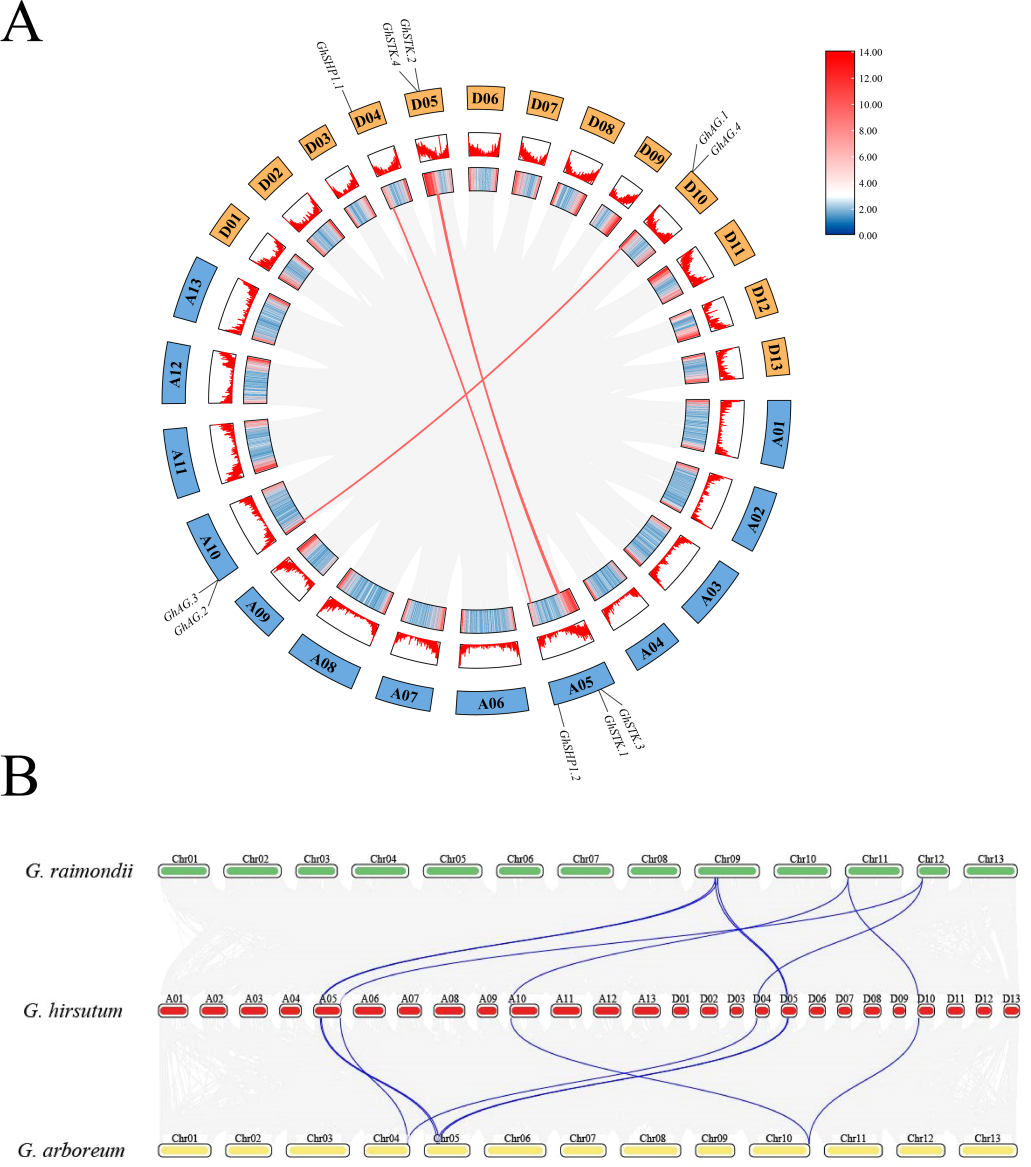
Intraspecific and interspecies replication analysis of *AG* subfamily genes. **(A)** Patterns of *GhAG* subfamily gene duplication in the genome of upland cotton. **(B)** Collinearity analysis of the three cotton species. *G. raimondii* is shown in green, *G. hirsutum* is shown in red, and *G. arboreum* is shown in yellow.

To investigate the expansion pattern of the AG subfamily in the three *Gossypium* species, collinearity analysis of the *AG* subfamily genes was performed. The results revealed four homologous duplicate gene pairs in upland cotton (**Figure 2A**), three of which were tandem duplications and one of which was a segmental duplication. The *Ka: Ks* ratios of the *GhAG* members were less than 0.30, suggesting that purifying selection has played a significant role in the evolutionary processes of genes within the *GhAG* subfamily (**Supplementary Table S3**). In addition, we found that most *GhAG* genes presented a collinear relationship with the *GaAG* and *GrAG* genes (**Figure 2B**).

### 3.4 Analysis of *cis*-acting elements in the promoters of *GhAG* subfamily genes

**Figure 3:**
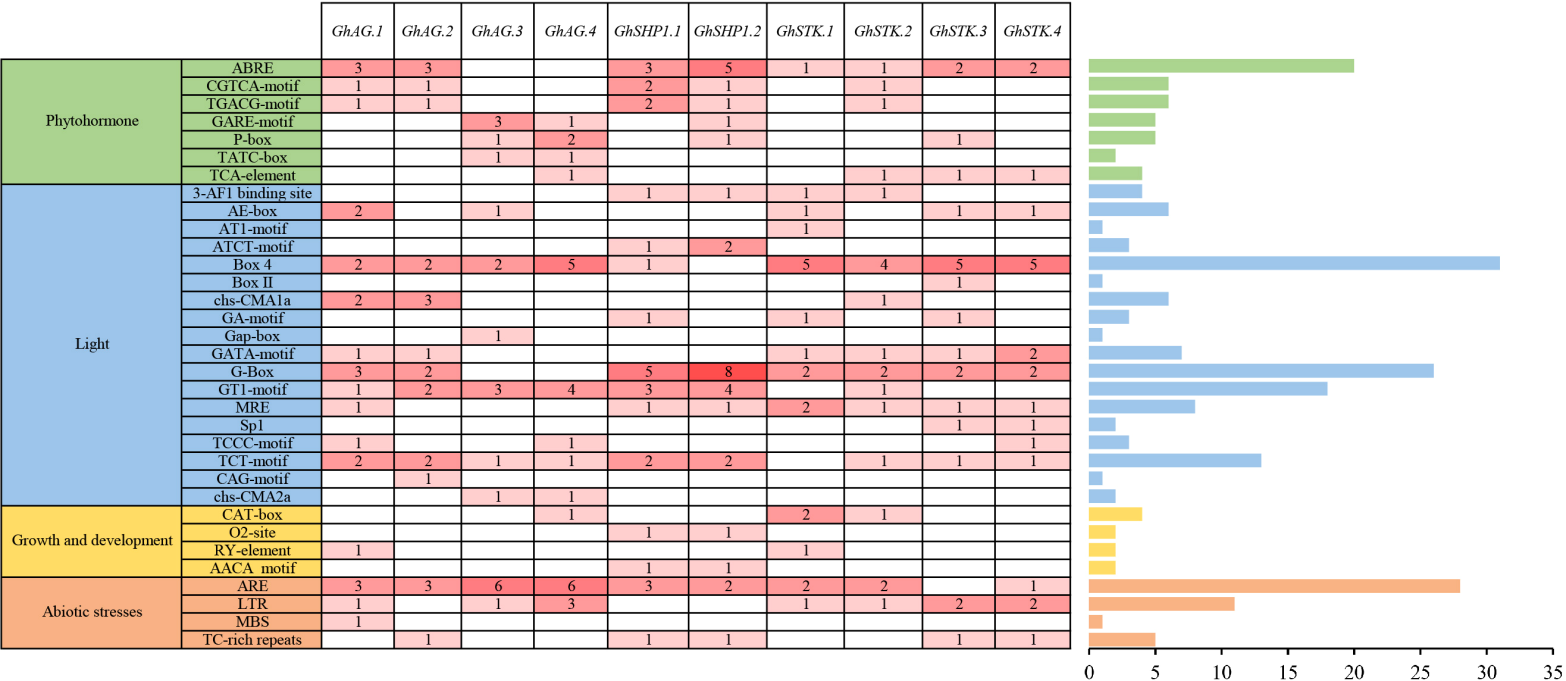
*Cis*-acting elements located within the promoter regions of *GhAG* genes. The Arabic numerals in the cells denote the quantity of *cis*-acting elements present.

To predict the potential biological functions of the *GhAG* subfamily genes, we analyzed the *cis*-acting elements present in their promoters. The *cis*-acting elements associated with *GhAGs* can be classified into four primary categories: phytohormone-responsive elements, light-responsive elements, plant growth and development-responsive elements, and abiotic stress-responsive elements (**Figure 3**). Among these categories, light-responsive elements constituted the majority of the *cis*-regulatory components within the promoters of the *GhAGs*, whereas elements responsive to plant growth and development constituted the smallest proportion. Among the light-responsive elements, Box 4 (31) was the most common and was present in the promoters of almost all the *GhAG* genes. Furthermore, a total of 20 ABREs associated with the ABA hormone response and 28 ARE elements related to the abiotic stress response were identified in the promoters of the *GhAGs*. CAT-box elements accounted for the largest number of elements responsive to growth and development, with only four. These findings indicate that *GhAGs* not only play a significant role in plant hormone regulation but are also associated with the response to abiotic stress.

### 3.5 Tissue-specific expression patterns of *GhAG* subfamily genes in upland cotton

**Figure 4:**
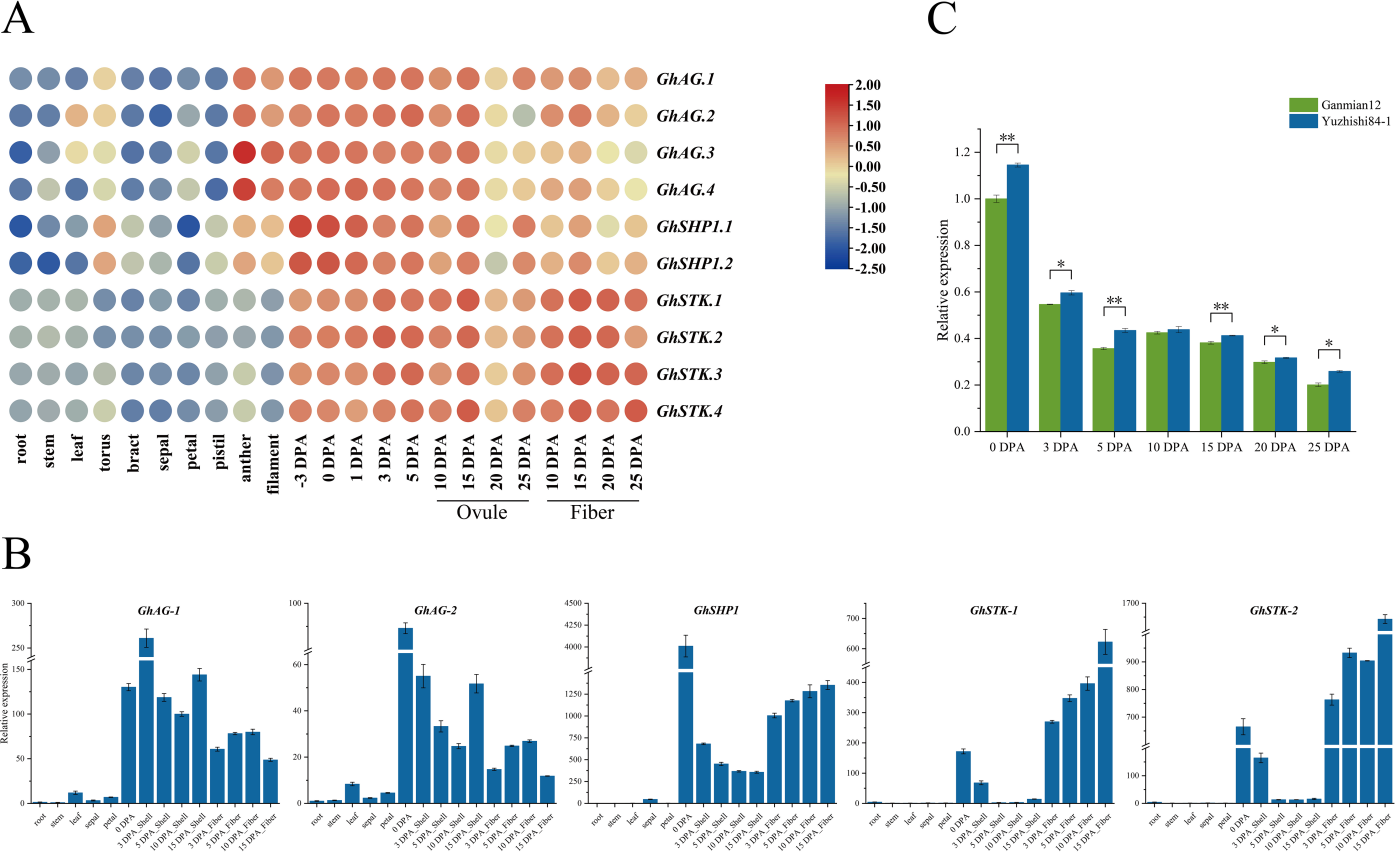
Analysis of the expression patterns of *GhAG* subfamily genes across various tissues in upland cotton. **(A)** Expression patterns of *GhAG* subfamily genes in different tissues of TM-1. The color of the continuous gradation represents the expression level. Red represents high expression, and blue represents low expression. **(B)** Expression patterns of *GhAG* subfamily genes in different tissues of ZM113. **(C)** *GhSHP1* expression in the cotton boll shell tissues of early and late opening boll varieties.

The expression patterns of genes are closely linked to their biological functions, which leads to contrasting features between species (Hu et al., 2019). To investigate the expression patterns of *GhAG* subfamily genes, we used FPKM values from transcriptome data to generate heatmaps to characterize gene expression levels across various tissues. The transcriptome data of different TM-1 tissues included data from root, stem, leaf, torus, bract, sepal, petal, pistil, anther, filament, ovule and cotton fiber tissues (**Figure 4A**). We found that *GhAG* subfamily genes presented relatively low expression levels in vegetative tissues, such as roots, stems and leaves, and presented three types of expression patterns in reproductive organs, such as flowers and fruits (**Figure 4A**). *GhAG.1*, *GhAG.2*, *GhAG.3* and *GhAG.4* expression was consistent with the type I pattern, and these genes were expressed mainly in the anthers and filaments and during the early stage of ovule and fiber development (-3 to 15 DPA). The *GhSHP1.1* and *GhSHP1.2* expression patterns were type II, with high expression occurring mainly during early ovule and fiber development (-3 to 15 DPA), especially three days before flowering and on the day of flowering. The *GhSTK.1*, *GhSTK.2*, *GhSTK.3* and *GhSTK.4* expression patterns were type III, with high expression mainly in ovules (15 DPA) and fibers during the late development period (15 to 25 DPA). It follows that *GhAG* subfamily genes are highly likely to affect floral organs as well as the development of later ovules and fibers.

Given that the above transcriptome data did not include the expression of *GhAG* subfamily genes in cotton boll shells, we further identified the expression patterns of *GhAGs* across various tissues of ZM113 (roots, stems, leaves, sepals, petals, cotton boll shells and fibers) using qRT−PCR. The qRT−PCR primers were not effective in distinguishing between *GhAG.1* and *GhAG.2*, *GhAG.3* and *GhAG.4*, *GhSHP1.1* and *GhSHP1.2*, *GhSTK.1* and *GhSTK.2*, and *GhSTK.3* and *GhSTK.4*. Therefore, we named the above gene pairs *GhAG-1*, *GhAG-2*, *GhSHP1*, *GhSTK-2* and *GhSTK-1* and detected their expression in different tissues of ZM113 (**Figure 4B**). We found that *GhSHP1* was highly expressed in both cotton boll shell and fiber tissues during the later stage of growth and development and that *GhSHP1* transcript levels were greater than that of *GhAG-1*, *GhAG-2*, *GhSTK-1* and *GhSTK-2* in cotton boll shells. After comprehensive analysis, *GhSHP1* was selected as the target gene.

To further verify whether *GhSHP1* affects cotton boll cracking, qRT−PCR was used to study the difference in *GhSHP1* expression in the cotton boll shell tissues of early (Yuzhishi84-1) and late (Ganmian12) opening bolls varieties at different growth stages and during boll shell cracking (**Figure 4C**). The results revealed that *GhSHP1* expression in the cotton boll shell of Yuzhishi84-1 was greater than that in the boll shell of Ganmian12. The difference is significant at 0, 3, 5, 15, 20 and 25 DPA. In summary, we hypothesized that *GhSHP1* may play a regulatory role in the development of cotton boll shells.

### 3.6 *GhSHP1* silencing leads to delayed flowering and cracking of cotton bolls

**Figure 5:**
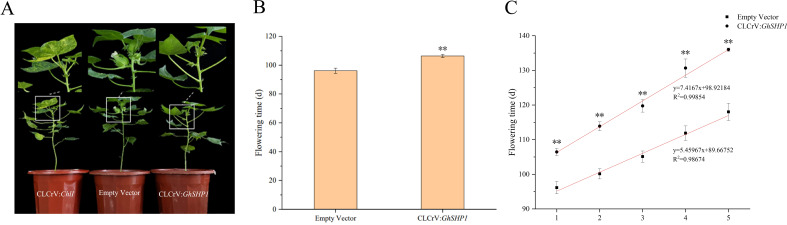
Phenotypic analysis of VIGS-treated upland cotton at the flowering stage. **(A)** Flowering phenotypes of plants treated with CLCrV: *ChlI*, empty vector, or CLCrV: *GhSHP1*. **(B)** Statistical analysis of the first flowering time of upland cotton. **(C)** Statistical analysis of the first five flowering times of upland cotton.

To assess the functions of *GhSHP1*, we employed VIGS technology to suppress its expression. Approximately 21 days after viral infection, the CLCrV: *ChlI* positive control plants were yellow, indicating that virus-induced silencing was successful in upland cotton plants (**Figure 5A**). The first flowering time, first five flowering times and cracking time of the silenced CLCrV: *GhSHP1* plants and empty vector plants were analyzed statistically. We found that the flowering time of the CLCrV: *GhSHP1* plants was approximately 10.3 days later than that of the empty vector plants (**Figure 5B**). The flowering times of the first five flowers were measured to analyze whether silencing the *GhSHP1* gene affected the flowering concentration of upland cotton. The scatterplot of the flowering times of the first five flowers was analyzed using linear regression, and the results revealed that silencing *GhSHP1* caused the flowering time of the upland cotton plants to be less concentrated (**Figure 5C**). These findings indicated that *GhSHP1* gene silencing not only delayed the flowering time of upland cotton but also reduced the flowering concentration.

**Figure 6:**
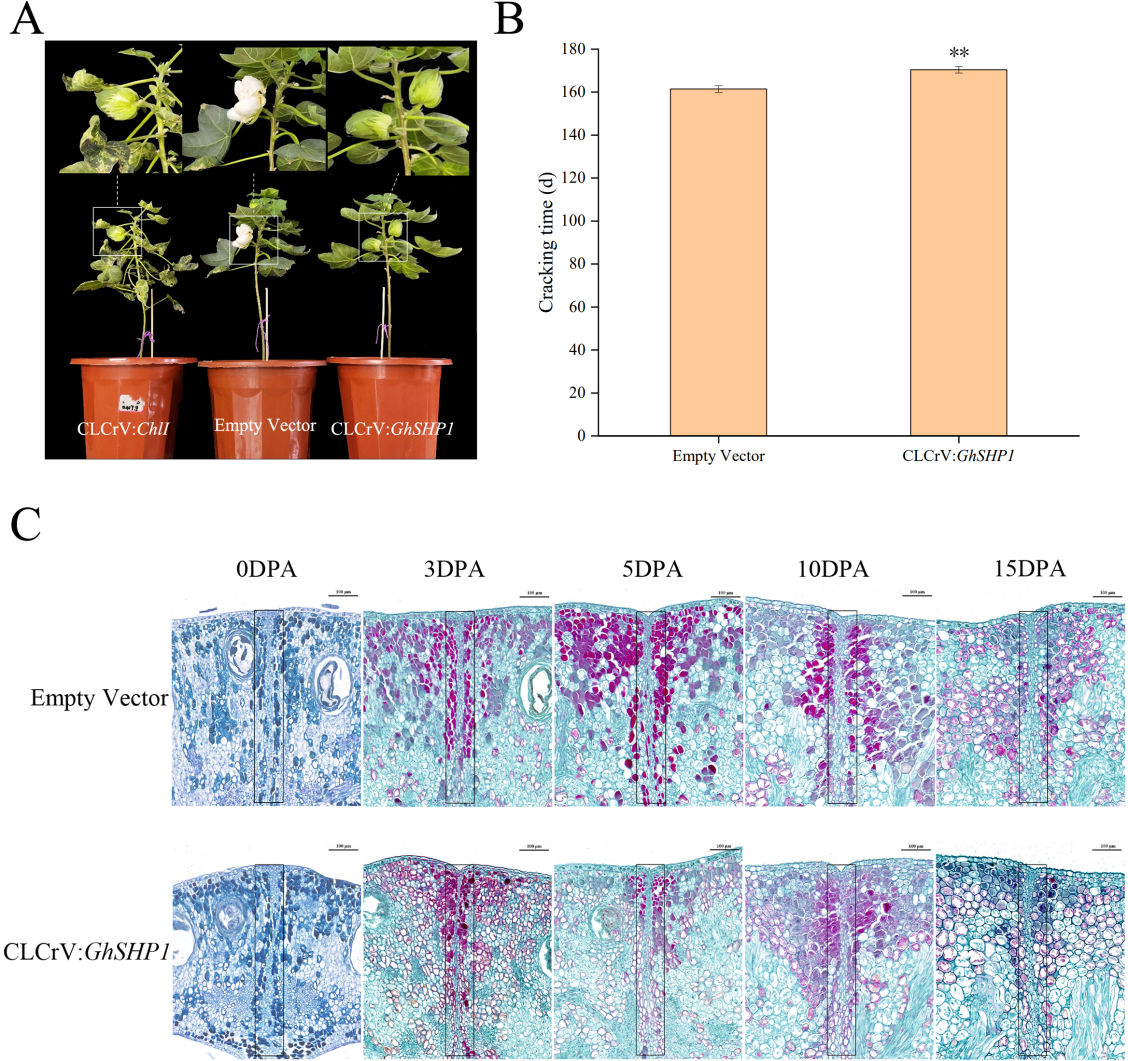
Phenotype and paraffin section analysis of upland cotton during the boll opening period. **(A)** Cotton boll cracking phenotypes of plants treated with CLCrV: *ChlI*, empty vector, or CLCrV: *GhSHP1*. **(B)** Statistical analysis of the boll cracking time of upland cotton. **(C)** Paraffin sections of cotton boll shell back sutures from empty vector-transformed plants and CLCrV: *GhSHP1*-silenced plants. The dehiscence zones are marked with a black frame. Scale bar = 100 μm.

Further observations indicated that the boll opening time of the CLCrV: *GhSHP1*-silenced cotton plants was approximately 9.0 days longer than that of the empty vector control plants (**Figures 6A, B**). Paraffin sections of cotton bollback sutures collected at different time points were observed under a microscope. The factors influencing the cracking time of the cotton bolls were analyzed from a cytological point of view (**Figure 6C**). In this image, the red cells are lignified cells colored with safranin O. On the left and right are the valves, and in the middle of them are the valve margins and the dehiscence zone (DZ). The outermost rectangular layer of cells in the valve is the ectocarp, and the inside layer is the mesocarp. During the early stage of fruit development, the DZ between adjacent valves is composed of numerous small square cells closely arranged in a line. However, until 3 DPA, the formation of such compact small cells could not be clearly observed in the CLCrV: *GhSHP1*-silenced plants. By examining paraffin sections of cotton boll shells at 0, 3, 5, 10, and 15 DPA, we observed that the DZ cells in the CLCrV: *GhSHP1*-silenced plants were thinner than those in the empty vector plants. On the other hand, at 3 DPA, unique lignified cells were observed on the back sutures of the cotton boll in both the empty vector plants and the silenced plants. At 5, 10 and 15 DPA, the plants transformed with empty vector exhibited more lignified cells than did the silenced plants. Near the DZ is the valve margin, where the lignification of cells and the inner cell layer of the valve results in pod shattering (Giménez et al., 2010). As the fruit ripens and dries, the parenchyma cells of the cotton boll valve shrink, creating tension in the hard lignified area, which helps the DZ shatter (Liljegren et al., 2000). This could cause the bolls of the empty vector-transformed plants to crack earlier than those of the CLCrV: *GhSHP1*-silenced plants.

### 3.7 Effects of *GhSHP1* silencing on key genes involved in fruit shattering

**Figure 7:**
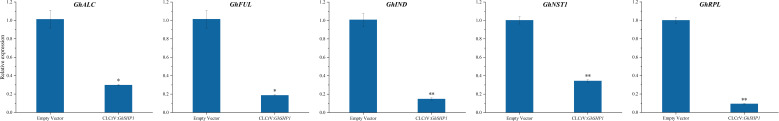
Quantitative analysis of key genes associated with fruit shattering in both control plants and *GhSHP1*-silenced plants. Asterisks denote significant differences in the expression levels of related genes between silenced and control plants (*P<0.05, **P<0.01).

*ALCATRAZ* (*ALC*), *FRUITFULL* (*FUL*), *INDEHISCENT* (*IND*), *NAC SECONDARY WALL THICKENING PROMOTING FACOTR1* (*NST1*) and *REPLUMLESS* (*RPL*) are key genes involved in regulating pod shattering in *A. thaliana* (Dong and Wang, 2015). *GhALC*, *GhFUL*, *GhIND*, *GhNST1* and *GhRPL* expression characteristics were examined in both the CLCrV: *GhSHP1*-silenced plants and the control plants. The expression levels of these five genes in the control plants were markedly greater than those in the *GhSHP1*-silenced plants (**Figure 7**). These results indicated that *GhSHP1* silencing strongly affected the expression of cracking-related genes, indicating that *GhSHP1* could be an important gene in the cotton boll opening regulatory network.

## 4 Discussion

In plants, fruit cracking is affected by fruit size, shape, growth rate, water content, skin characteristics, internal fruit cracking-related gene expression and external factors such as temperature, light, and precipitation (Hu et al., 2024). These factors ultimately act on the outer peel, which is unable to withstand the expansion force from inside the peel and cracks (Correia et al., 2018; Khadivi-Khub, 2015; Li et al., 2021). The split fruit can also be subdivided into the following four fruit types: silique, legume, capsule, and follicle (Hernandez et al., 2023). The fruit of *A. thaliana* is a silique that develops from two symbiotic carpels, with a lateral membranous placenta and a replum that is generated at the ventral suture of the carpel to divide the ovary into two chambers. When the siliques are ripe, the peel cracks along both sides of the abdominal suture and falls off in two pieces. The fruit of soybean is the legume, and the pod is the fruit formed by the development of a single carpel. At maturity, it splits along the abdominal suture and the back suture simultaneously, and the peel splits into two pieces. The fruit of cotton is the capsule. The cotton boll develops from a compound pistil of a conjunctive carpellary and has an axial placenta. It is usually formed by the carpel, with three to five chambers. The fruit ripens and cracks in a way known as loculicidal dehiscence, along the sutures of the back. Follicles develop from a single carpellary pistil or from the apocarpous gynoecium. When ripe, the fruit will split along one side of the dorsal or abdominal suture.

Members of the MADS-box family are integral to various aspects of plant biology, including flower and seed development, the regulation of flowering time, fruit maturation processes, and responses to both abiotic and biotic stresses (Schilling et al., 2018). To date, genes belonging to this family have been reported in *A. thaliana*, rice, soybean, tomato and other plants. The target genes identified in this study are classified within the AGAMOUS subfamily of the MADS-box family, and bioinformatics analysis of the identified *GhAGs* was performed. Ten *AG* subfamily genes were identified in upland cotton. This result is consistent with a report from Ren et al (Ren et al., 2017). The greatest number of *AGs* was detected in *Brassica napus* (15) (Wu et al., 2018). This was followed by the tetraploid cotton *G. hirsutum* (10) and *G. barbadense* (10), as well as the legume crop *Glycine max* (10) (Nardeli et al., 2018; Shu et al., 2013). Furthermore, phylogenetic analyses revealed that *AG* subfamily genes have undergone a series of genome amplification events during the evolutionary transition of cotton from diploid to tetraploid forms. This process doubled the number of *AG* subfamily genes within the allotetraploid cotton species *G. hirsutum* and *G. barbadense* compared with *G. raimondii* and *G. arboreum*. *AtSHP2* homologous genes were found only in *Bn*, *Br* and *Cr* but not in the other 11 species. This may be due to gene loss that occurred during the evolutionary process. In this study, four *AG* subfamily genes were identified in *Oryza sativa*, whereas five *OsAGs* were identified by Ren et al (Ren et al., 2017). This discrepancy may arise from the utilization of different reference genomes or from inconsistencies in the screening criteria employed. These findings suggested that the *AG* subfamily genes share a common origin and that different species evolved different numbers of *AG* subfamily genes during evolution.

Most *AG* subfamily genes in upland cotton presented one-to-one collinearity. A good collinearity relationship was noted with the other two cotton species. The *Ka: Ks* ratios for all the members of the *GhAG* subfamily were less than 1. *Ka*/*Ks* values can determine whether selection pressure is acting on the gene that encodes the protein. If *Ka*/*Ks* is > 1, it indicates a positive selection effect. If *Ka*/*Ks* = 1, this suggests neutral selection. If *Ka*/*Ks* is < 1, it is indicative of purifying selection. These findings indicate that purifying selection has played a significant role in the evolutionary development of *GhAG* subfamily genes. An examination of the *cis*-acting elements within promoters revealed that the majority of them were light-responsive elements, and all of them were located in the nucleus. Taken together with the results of the expression pattern analysis, these findings suggest that *GhAGs* are likely to play regulatory roles in plant growth and development by affecting flower organs and fruits. This is consistent with findings from prior research (Dreni et al., 2011; Liu et al., 2016, 2018; Lu et al., 2019; ÓMaoiléidigh et al., 2013; Xu et al., 2017).

To further investigate the effect of *GhSHP1* on cotton boll cracking, we conducted a VIGS experiment. Compared with those of the control plants, the flowering and boll cracking times of the *GhSHP1*-silenced plants were delayed. Paraffin sections were prepared at the suture line on the posterior side of the cotton boll. The findings indicated that the development of the DZ in the silenced plants occurred later than that observed in the control plants during the same timeframe; furthermore, the extent of valve lignification was lower in the silenced plants than in the control plants. *GhSHP1* may affect cotton boll cracking by regulating DZ development. Research has demonstrated that, in *A. thaliana*, *AtSHP1/2* plays a role in promoting the differentiation of the DZ while simultaneously promoting the lignification of adjacent cells (Liljegren et al., 2000). In *Brassica napus*, BnSHP^5^-184 with five homologous mutants was obtained using the CRISPR-Cas9 system, and fewer lignification and separation layers were found in and around the valve. *BnSHP1A09* may be a promising site for controlling the DZ lignin content (Zaman et al., 2021). In fleshy tomato fruits, the *AtSHP1/2* homologous gene *TOMATO AGAMOUS-LIKE1* (*TAGL1*) promotes fruit ripening (Itkin et al., 2009; Jeon et al., 2024; Vrebalov et al., 2009). *TAGL1* overexpression in *A. thaliana* leads to a phenotype that closely resembles the phenotype observed with *SHP1/2* overexpression. The functions of *SHP* in these species are similar, and there is no difference in function due to different fruit types. These findings suggest that the role of *SHP1/2* genes in determining organ identity is fundamentally conserved (Pinyopich et al., 2003).

Fruit dehiscence is caused by a combination of many factors in the plant, which form a complex regulatory network. To further investigate the role of *GhSHP1* in the regulatory network of cotton boll cracking, we selected five key genes in the tissue differentiation regulatory network required for *A. thaliana* pod fragmentation. In this study, qRT−PCR analysis revealed a significant decrease in the expression levels of the five genes in plants with silenced *GhSHP1*. Therefore, a positive regulatory relationship may exist between *GhSHP1* and these five genes. In contrast, *FUL* exerts a negative regulatory effect on *SHP1/2* within the regulatory network governing pod cracking in *A. thaliana* (Ferrándiz et al., 2000). In *FUL1/2* RNAi-treated fruits, the expression of the tomato gene *TAGL1* was upregulated in the peel, indicating a negative regulatory relationship between *FUL1/2* and *TAGL1* (Bemer et al., 2012). Moreover, for key genes in the *A. thaliana* pod-shattering regulatory pathway, we predicted the protein interaction network of upland cotton homologous genes (**Supplementary Figure S1**). These findings indicate that *GhSHP1* expression may be directly correlated with *GhIND* and *GhRPL* expression. Therefore, *GhSHP1* may have an indirect regulatory relationship with both *GhFUL* and *GhALC*. This may explain why the expression levels of these four genes differed significantly between the *GhSHP1*-silenced plants and the control plants.

## 5 Conclusion

In summary, we identified 10, 10, 5 and 5 *AGAMOUS* subfamily genes in *G. hirsutum*, *G. barbadense*, *G. raimondii* and *G. arboreum*, respectively. We conducted bioinformatic analyses of the *GhAGs*, including phylogenetic studies, collinearity assessments, repeated event evaluations, investigations of promoter *cis*-acting elements, and expression pattern analyses. The *GhAG* promoter sequences include numerous light-responsive elements as well as elements associated with plant hormones. Expression pattern analysis revealed that *GhAGs* may affect the development of flower organs, ovules, cotton boll shells and fibers during the later stages of growth and development. In addition, on the basis of the VIGS experiment results, we concluded that silencing the *GhSHP1* gene led to delayed flowering and boll opening in upland cotton. The results of the paraffin section analysis indicate that *GhSHP1* may affect the cracking time of cotton bolls by influencing the development of the DZ at the back suture line of the cotton boll shell. This study provides a reference for future studies of key genes affecting cotton boll cracking and for the molecular breeding of early-maturing cotton varieties.

## Data Availability

The original contributions presented in the study are included in the article/**Supplementary Material**. Further inquiries can be directed to the corresponding authors.

## Supplementary material

Supplementary Table 1 Physicochemical properties of the AG subfamily members in upland cotton.

Supplementary Table 2 Species information in phylogenetic evolutionary trees.

Supplementary Table 3
*Ka/Ks* analysis of *AG* subfamily genes in upland cotton.

Supplementary Figure 1 Interaction network between the *GhSHP1* protein and key shatter-related proteins.
