# Supplementary material for: Silencing of GhSHP1 hindered flowering and boll cracking in upland cotton
Source: Front Plant Sci. 2025 Feb 25;16:1558293. doi: 10.3389/fpls.2025.1558293 (PMC11893620; doi:10.3389/fpls.2025.1558293)
Supplement: Supplementary Table 1 — Physicochemical properties of the AG subfamily members in upland cotton. [file Table1.docx]

Table S1. Protein physicochemical properties of AG subfamily members in upland cotton

| **Gene ID** | **Gene Name** | **Number of Amino Acids(aa)** | **Melecular Weight**  **(kDa)** | **Theoretical pI** | **Instability Index** | **Aliphatic Index** | **Grand Average of Hydropathicity** | **Subcellular Localization** |
| --- | --- | --- | --- | --- | --- | --- | --- | --- |
| *GH_A05G2521* | *GhSTK.3* | 223 | 25.73 | 9.44 | 55.15 | 90.54 | －0.601 | nucleus |
| *GH_A05G2747* | *GhSTK.1* | 224 | 25.82 | 9.43 | 48.47 | 86.65 | －0.622 | nucleus |
| *GH_A05G3983* | *GhSHP1.2* | 234 | 26.97 | 9.41 | 56.18 | 81.32 | －0.726 | nucleus |
| *GH_A10G0346* | *GhAG.2* | 246 | 28.37 | 9.25 | 56.34 | 77.72 | －0.848 | nucleus |
| *GH_A10G0347* | *GhAG.3* | 246 | 28.27 | 9.36 | 62.44 | 78.94 | －0.801 | nucleus |
| *GH_D04G0393* | *GhSHP1.1* | 234 | 26.99 | 9.41 | 55.86 | 79.66 | －0.737 | nucleus |
| *GH_D05G2543* | *GhSTK.4* | 223 | 25.72 | 9.44 | 55.89 | 88.79 | －0.625 | nucleus |
| *GH_D05G2764* | *GhSTK.2* | 224 | 25.82 | 9.43 | 48.47 | 86.65 | －0.622 | nucleus |
| *GH_D10G0360* | *GhAG.1* | 246 | 28.35 | 9.34 | 58.91 | 77.72 | －0.848 | nucleus |
| *GH_D10G0361* | *GhAG.4* | 246 | 28.28 | 9.36 | 62.03 | 77.76 | －0.830 | nucleus |
