# Supplementary material for: Silencing of GhSHP1 hindered flowering and boll cracking in upland cotton
Source: Front Plant Sci. 2025 Feb 25;16:1558293. doi: 10.3389/fpls.2025.1558293 (PMC11893620; doi:10.3389/fpls.2025.1558293)
Supplement: Supplementary Table 2 — Species information in phylogenetic evolutionary trees. [file Table2.docx]

Table S2. Species information in phylogenetic evolutionary trees

| Fruit type | Species | Short name | Gene number |
| --- | --- | --- | --- |
| Capsule | *Gossypium hirsutum* | *Gh* | 10 |
|  | *Gossypium barbadense* | *Gb* | 10 |
|  | *Gossypium raimondii* | *Gr* | 5 |
|  | *Gossypium arboreum* | *Ga* | 5 |
| Siliqua | *Arabidopsis thaliana* | *At* | 4 |
|  | *Brassica napus* | *Bn* | 15 |
|  | *Brassica rapa ssp. pekinensis* | *Br* | 8 |
|  | *Brassica oleracea var. capitata* | *Bo* | 6 |
|  | *Capsella rubella* | *Cr* | 4 |
| Legume | *Glycine max* | *Gm* | 10 |
|  | *Arachis hypogaea* | *Ah* | 7 |
|  | *Medicago truncatula* | *Mt* | 4 |
|  | *Cercis canadensis* | *Cc* | 3 |
| Caryopsis | *Oryza sativa* | *Os* | 4 |
| Drupe | *Theobroma cacao* | *Tc* | 3 |
