# Supplementary material for: Silencing of GhSHP1 hindered flowering and boll cracking in upland cotton
Source: Front Plant Sci. 2025 Feb 25;16:1558293. doi: 10.3389/fpls.2025.1558293 (PMC11893620; doi:10.3389/fpls.2025.1558293)
Supplement: Supplementary Table 3 — Ka/Ks analysis of AG subfamily genes in upland cotton. [file Table3.docx]

Table S3. *Ka/Ks* analysis of *AG* subfamily genes in upland cotton

| gene 1 | gene 2 | *Ka* | *Ks* | *Ka/Ks* |
| --- | --- | --- | --- | --- |
| GH_A05G3983 | GH_D04G0393 | 0.00185 | 0.00625 | 0.295929 |
| GH_A05G2521 | GH_D05G2543 | 0.00193 | 0.027122 | 0.071157 |
| GH_A05G2747 | GH_D05G2764 | 0 | 0.053178 | 0 |
| GH_A10G0346 | GH_D10G0360 | 0.003473 | 0.038265 | 0.090767 |
