# Supplementary figures and images for: Silencing of GhSHP1 hindered flowering and boll cracking in upland cotton
Source: Front Plant Sci. 2025 Feb 25;16:1558293. doi: 10.3389/fpls.2025.1558293 (PMC11893620; doi:10.3389/fpls.2025.1558293)

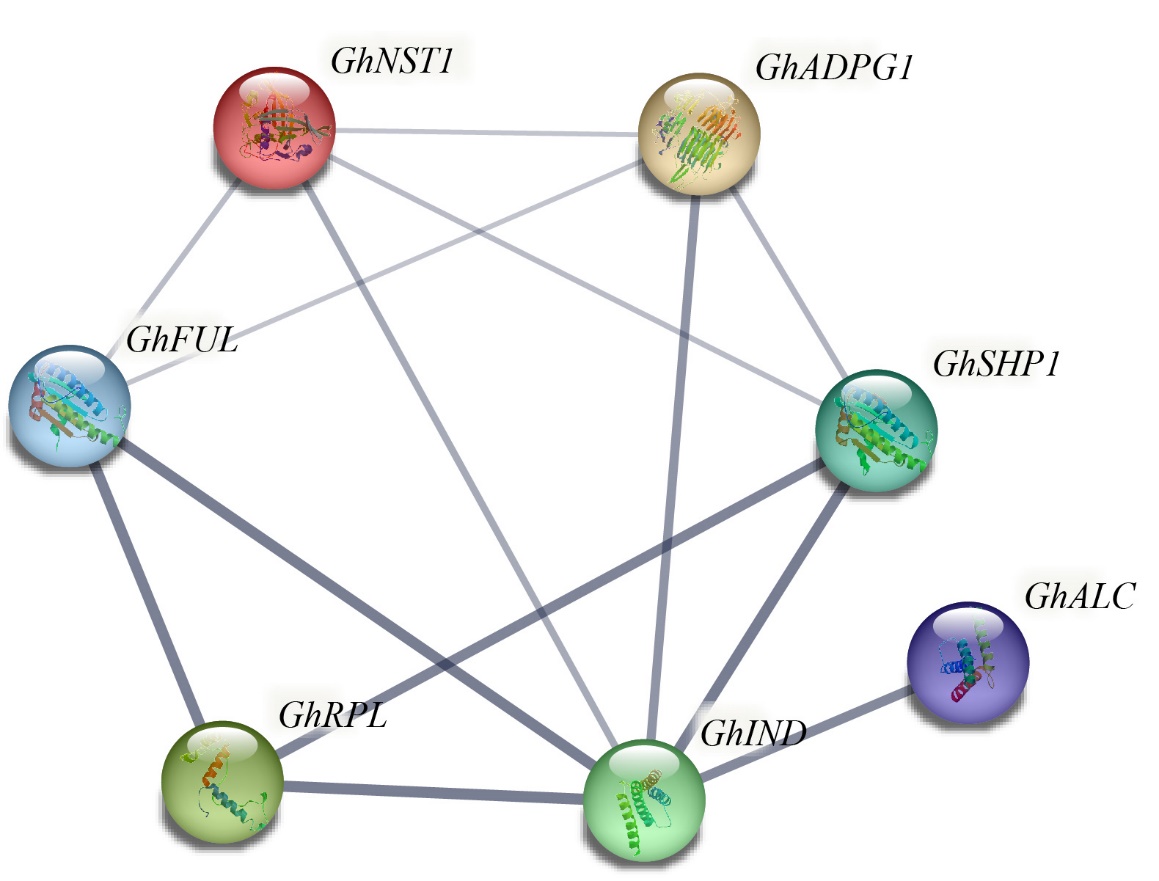


Figure S1. Interaction network between *GhSHP1* protein and key shattering related proteins.

Supplement: Supplementary Figure 1 — Interaction network between the GhSHP1 protein and key shatter-related proteins. [file DataSheet1.docx]
